# Is neonatal uterine bleeding responsible for early-onset endometriosis?

**DOI:** 10.1186/s12958-023-01099-1

**Published:** 2023-06-19

**Authors:** Kanae Ogawa, Khaleque N Khan, Haruo Kuroboshi, Akemi Koshiba, Koki Shimura, Tatsuro Tajiri, Shigehisa Fumino, Hiroyuki Fujita, Tomoharu Okubo, Yoichiro Fujiwara, Go Horiguchi, Satoshi Teramukai, Akira Fujishita, Kyoko Itoh, Sun-Wei Guo, Jo Kitawaki, Taisuke Mori

**Affiliations:** 1 Department of Obstetrics and Gynecology, Graduate School of Medical Science, Kyoto Prefectural University of Medicine, Kyoto, Japan; 2 The Clinical and Translational Research Center, Graduate School of Medical Science, Kyoto Prefectural University of Medicine, Kyoto, Japan; 3 Department of Pediatric Surgery, Graduate School of Medical Science, Kyoto Prefectural University of Medicine, Kyoto, Japan; 4 Present Address: Department of Pediatric Surgery, Faculty of Medical Sciences, Kyushu University, Fukuoka, Japan; 5 Japanese Red Cross Society Kyoto Daini Hospital, Kyoto, Japan; 6 Japanese Red Cross Society Kyoto Daiichi Hospital, Kyoto, Japan; 7 Kyoto City Hospital, Kyoto, Japan; 8 Department of Biostatistics, Graduate School of Medical Science, Kyoto Prefectural University of Medicine, Kyoto, Japan; 9 Department of Gynecology, Saiseikai Nagasaki Hospital, Nagasaki, Japan; 10 Department of Pathology and Applied Neurobiology, Graduate School of Medical Science, Kyoto Prefectural University of Medicine, Kyoto, Japan; 11 Shanghai Obstetrics and Gynecology Hospital, Fudan University, Shanghai, China

**Keywords:** Neonatal uterine blood, Neonatal endometrium, eMSCs, Immunocompetent cells, NUB hypothesis, Early-onset endometriosis

## Abstract

**Background:**

It has been hypothesized that the origin of early-onset endometriosis could be from endometrial mesenchymal stem cells (eMSCs) in neonatal uterine blood (NUB). There is no information on the possible mechanistic basis linking an association between NUB/neonatal endometrium and development of early-onset endometriosis. In this study we performed a series of experiments to clarify the mechanistic link between NUB and/or neonatal endometrium and development of early-onset endometriosis.

**Methods:**

We retrospectively collected postmortem neonatal endometria (n = 15) and prospectively collected NUB (n = 18) of female babies for the analysis of different biological markers including eMSCs. Immunohistochemical analysis of neonatal endometria was performed to examine the expression patterns of ovarian steroid receptors (ER/PGR), decidualization (prolactin, IGFBP1), pre-decidualization (Glycodelin A, α-SMA), proliferation (Ki-67 index), vascularity (CD31 + cells), immunocompetent CD68+, CD45+, CD56 + cells and some putative markers of eMSCs. Cell transfer method and immunocytochemistry were used to investigate the eMSCs and/or endometrial cells in NUB.

**Results:**

Immunohistochemical analysis of postmortem neonatal endometria revealed variable staining response to ER/PGR, decidual markers, and substantial proliferative and angiogenic activity. A moderate to strong immunoexpression of Glycodelin-A was found in both neonatal and adult endometria. The tissue infiltration of CD56+, CD45 + and CD68 + immunocompetent cells was significantly low in neonatal endometria than that in adult endometria (p = 0.0003, p < 0.0001, p = 0.034, respectively). No eMSCs or even endometrial cells were detected in NUB. However, a variable expression of some phenotypes of eMSCs (CD90/CD105) was found in neonatal endometria.

**Conclusions:**

Based on our serial experiments we did not find any supporting evidence for the role of NUB in early-onset endometriosis. Neonatal endometria showed variable expression of ovarian steroid receptors, decidualization, and a substantial amount of proliferative and angiogenic activity. As an alternative mechanism, a significantly less tissue accumulation of immunocompetent cells in neonatal endometria may explain the survival of ER + and PGR + cells should they make entry into the pelvis and consequent development of early endometriosis with the onset of ovarian function. Future study with large sample size and application of modified technological tools is warranted to test the NUB hypothesis and to clarify their biological or clinical significance.

**Trial registration:**

not applicable.

**Supplementary Information:**

The online version contains supplementary material available at 10.1186/s12958-023-01099-1.

## Introduction

Endometriosis is an estrogen-dependent chronic inflammatory disease affecting mostly women of reproductive age. First described over three centuries ago, endometriosis is classically defined as the presence of endometrial glands and stroma in extrauterine locations [1]. Endometriosis is a multifactorial condition and is difficult to uniformly explain its pathogenesis by a single factor. There is no consensus concerning the histologic origin of endometriosis. Although retrograde menstruation and/or coelomic metaplasia are the widely accepted hypotheses of its pathogenesis [2, 3], cases of pre-menarcheal endometriosis are apparently incongruent with these hypotheses. As such, the pathogenesis of pre-menarcheal endometriosis may differ from endometriosis in adolescent girls and adult women. It has been proposed recently that mesenchymal stem cells of endometrial origin (eMSCs) may contribute to the occurrence of early endometriosis when transported to the pelvis [4, 5]. However, to the best of our knowledge, no study has been conducted so far to test this hypothesis.

To account for early-onset endometriosis, one hypothesis that neonatal uterine bleeding (NUB) may be responsible has been proposed. The hypothesis posits that, like retrograde menstruation in adult women, NUB that contains eMSCs can also be regurgitated into the peritoneal cavity, remains dormant for years and then is reactivated by the rising estrogen levels during thelarche or menarche and develops into early-onset endometriosis through a process of neo-angiogenesis under the influence of estrogens [6–10]. To further evaluate this hypothesis, we recently found that NUB occurs in neonates on day 1–8 with a prevalence rate of 3.1%. In addition, a web-based questionnaire survey indicated that young women complain of various endometriosis-related symptoms who were born with NUB (Ogawa et al., unpublished data). Although NUB has been hypothesized to be the origin and progression of early-onset endometriosis [11–14], the exact underlying mechanistic basis is poorly described, and the evidence for the hypothesis is completely absent as of today.

The link between endometriosis and NUB was first reported in a postmortem examination of an infant suffering from McKusick-Kauffman syndrome, with an intact vaginal septum and a hemorrhagic endometrial reflux resulting in congenital endometriosis [15]. Despite this information in 1996, NUB is completely overlooked in clinical practice due to the belief that it is clinically inconsequential. NUB has been described in detail by various authors during the second half of the 19th century and until 2016 [16]. However, due to its perceived clinical insignificance, further information on NUB and its possible role in early-onset endometriosis remains largely unclear. As of now, it is completely unclear as whether NUB and/or neonatal endometrium contain endometrial stem/progenitor cells. Similarly, evidence is lacking as whether NUB can be truly transported into the abdominal cavity. To prove or refute the NUB hypothesis, carefully designed prospective studies are warranted.

The pioneering postmortem study of Ober and Bernstein documented a broader spectrum of progesterone responses in uteri from neonates who had died soon after birth [17]. This study demonstrated that despite low incidence, decidual transformation and endometrial shedding do occur in the neonatal uterus [17]. Since the publication of this report in 1955, there is no further study demonstrating maternal hormonal response, proliferative and angiogenic activity of postmortem neonatal endometrium, likely due to its perceived clinically inconsequential nature.

While NUB is hypothesized to contain eMSCs and lay dormant in pelvis for many years until “awakened” during puberty [8, 9], the published literatures indicate that all these eMSCs lack ovarian steroid receptors [6, 10]. In the absence of steroid hormonal receptors, it is therefore difficult to understand that eMSCs in NUB can differentiate into endometriosis-like tissues or cells under the influence of ovarian steroids during puberty. It is also unclear as why the dormant endometrial cells and/or eMSCs, if any, in pelvis are not removed by the immunocompetent cells until puberty. This issue has prompted us to evaluate alternative mechanism to establish a link, if any, between NUB/neonatal endometria and early-onset endometriosis.

Although the immunocompetent cells of the adult human endometrium are well characterized, little is known about the infiltration of immunocompetent cells in neonatal endometria [18]. According to published reports, the distribution of immunocompetent cells, such as CD45 + leukocytes, CD68 + macrophages, and CD56 + natural killer (NK) cells in neonatal endometria differs from that in adult endometria [18]. In adult endometria, these cells significantly increase in secretory phase. While NK cells play an important role for implantation and early pregnancy loss in adult endometria [19, 20], few or no CD56 + cells were found in neonatal/children or in fetal endometria [18]. Therefore, the distribution of immunocompetent cells in neonatal endometria and their possible involvement in the survival of endometrial cells may clarify the mechanistic link between neonatal endometrium and early-onset endometriosis. Because once these cells are transported to the pelvis by reflux of NUB may remain dormant without clearance until the onset of ovarian function during thelarche or menarche.

In an attempt to address all these unclear issues as mentioned above, this study was set out to investigate the followings: (1) To investigated the expression profiles of different biological markers (EpCAM/CD10/Ki-67/CD31), ovarian steroid receptors (ER/PGR) and decidual/pre-decidual markers (prolactin/IGFBP1/Gd-A/α-SMA) in postmortem neonatal endometria by immunohistochemistry, (2) To examine the tissue infiltration of different immunocompetent cells such as CD68 + macrophages, CD45 + pan-leukocytes, and CD56 + NK cells in neonatal endometria by immunohistochemistry, and to further compare the expression profiles of all these markers in neonatal and adult endometria. (3) Since Glycodelin-A (Gd-A) is reported to act as one of the immunosuppressive molecules in addition to behave as a pre-decidual marker [21–24], we were curious to know the association, if any, between Gd-A expression and tissue accumulation of immune cells in neonatal endometria, (4) To prospectively collect overt NUB and its morphological analysis with hematoxylin and eosin (H&E) stain or Papanicolaou (Pap) stain, (5) To identify different putative eMSCs markers (SUSD2/PDGFR-β/CD90/CD105) in NUB by immunocytochemical analysis, (6) To localize different eMSCs, if any, in neonatal and adult endometria by immunohistochemical analysis, (7) To appraise the evidence of NUB, if any, in the development of early-onset endometriosis.

## Materials and methods

### Study design

This was a combined retrospective and prospective case-controlled cohort study. The first part investigated the expression profiles of different biological markers and eMSCs in postmortem neonatal endometria and adult endometria in an attempt to understand any difference in the biological responses and/or the presence of eMSCs between these two groups of endometria. With this purpose, we used uterine and endometrial samples of female neonates and adult women that were collected from autopsy cases and cases with different benign gynecological diseases during surgery, respectively. The second part of this study comprised of prospective collection of NUB in female babies after birth in an attempt to identify any eMSCs in NUB as well as in postmortem neonatal endometria. All retrospective collection of postmortem neonatal endometria and prospective collection of NUB and adult endometria were conducted in accordance with the guidelines of the Declaration of Helsinki and with informed consent where appropriate. This study was approved by the Ethics Committee of the Institutional Review Board (IRB) of Kyoto Prefectural University of Medicine (IRB approval No. ERB-C-1445-1).

#### Collection of neonatal uterine blood and identification of eMSCs

In order to collect visible uterine blood from newborn female babies, we made contact with different affiliated hospitals of our university and explained the purpose of our study to the concerned doctors, nurses and midwives of respective hospitals. When midwives discovered NUB during changing of diapers, they collected the blood on the diaper or vulva with tweezers or cut the area of blood stained diaper, immerged in Dulbecco’s modified essential medium (DMEM) and stored in a refrigerator until processing. During the period of 2019–2021, we could collect visible uterine blood samples from 18 newborn female babies. All samples were collected in DMEM except one that was collected in 4% paraformaldehyde (PFA). The blood adhering to diaper was washed with DMEM and centrifuged (450 g for 5 min). The cell pellets were transferred and smeared onto a slide glass and fixed with cytology fixative spray. Histological examination was performed by hematoxylin and eosin (H&E) stain or Papanicolaou (Pap) stain.

The immunocytochemical analysis was performed using target antibodies against EpCAM, CD10, ER, PGR, Gd-A, PRL, IGFBP1, αSMA, and a number of eMSCs markers such as SUSD2, PDGFRβ, CD90, and CD105. We applied cell transfer method for this analysis with following procedures: We used materials that had been stained with the Pap method. The slides were soaked in xylene to remove the coverslips. The slides were covered with 1: 1 mixture of xylene and mounting medium and dried. After warming the slides in a warm water bath (60℃), we peeled off the sheet on which target cells were attached with a cutter blade. We cut the sheet into small rectangular pieces for the designated number of antibodies, attached each sheet to a new slide glass in a warm water bath (60℃) and dried. Finally, slides were soaked in xylene to remove mounting medium and to complete the process of cell transfer. After that, immunostaining was performed without antigen retrieval. A representative procedure of NUB collection and cell transfer method is shown in Suppl. Figure 1.

#### Collection of neonatal and adult endometria

We collected tissues blocks from 15 autopsy cases undergoing postmortem examination at the Department of Pathology of our University. These cases were born at the gestational age of 27–40 weeks and died 0–32 days after birth for different pathological conditions. All these neonates were delivered by either elective Caesarian section or normal vaginal delivery. One neonate died within uterus (stillbirth) and was vaginally delivered at the gestational age of 34 weeks and 6 days. Among many cases, neonatal endometria from 15 autopsy cases were selected based on H&E-stained slides of intact endometrium and myometrium as confirmed by one expert histopathologist (KI) of our university. As a positive control, we collected adult endometria and myometria from 13 women who underwent laparoscopic surgery for uterine fibroids at Saiseikai Nagasaki Hospital in Nagasaki, Japan. All these 13 women, aged 41.3 ± 3.5 (mean ± SD) had no hormonal therapy within three months before surgery. Adult endometrial samples were classified according to anamnestic and histological dating into early secretory (day 15–22, n = 4) and late secretory (day 23–32, n = 9) phases.

#### Antibodies

In order to understand the biological behavior of neonatal endometria and its difference with adult endometria, we performed immunohistochemical analysis using respective antibodies against target antigens as follows: epithelial cell adhesion molecule (EpCAM, marker of gland cells), CD10 (marker of stromal cells), estrogen receptor (ER), progesterone receptor (PGR), prolactin (PRL), Insulin like growth factor binding protein 1 (IGFBP1), Glycodelin-A (Gd-A, pre-decidual marker), α-smooth muscle actin (αSMA, pre-decidual marker), Ki-67 (cell proliferation marker), CD31 (vascular cell marker), CD68 (marker of macrophages), CD45 (marker of pan-leukocytes), CD56 (marker of natural killer (NK) cells). To identify endometrial mesenchymal stems cells (eMSCs) in neonatal and adult endometria/myometria, we also performed immunohistochemistry using respective antibodies against eMSCs markers such as Sushi domain containing 2 (SUSD2), platelet-derived growth factor receptor-beta (PDGFRβ), CD90, CD105. A complete list of primary antibodies, concentrations used for each antibody, clonality, name of manufacturing companies, and respective positive controls are shown in Table 1.

**Table 1:** List of antibodies used in our current study

	Catalog No.	Clonality	Host	Conc. used	Name of Company	Positive control
EpCAM (Ber-EP4)	M0804	Monoclonal	Mouse	1:200	Dako	endometrium
CD10 (56C6)	M7308	Monoclonal	Mouse	1:100	Dako	endometrium
ER (6F11)	NCL-L-ER-6F11	Monoclonal	Mouse	1:200	Novocastra	endometrium
PGR (1A6)	NCL-L-PGR	Monoclonal	Mouse	1:100	Novocastra	endometrium
Ki-67	NCL-L-Ki67-MM1	Monoclonal	Mouse	1:100	Novocastra	endometrium
CD31 (JC70A)	M0823	Monoclonal	Mouse	1:400	Dako	endometrium
Prolactin (PRL02)	MA5-11998	Monoclonal	Mouse	1:100	Invitrogen	decidua
IGFBP1	NBP2-33475	Polyclonal	Rabbit	1:100	Novus biological	decidua
Glycodelin-A	LS-B10557/ 73,691	Polyclonal	Rabbit	1:400	LifeSpan BioSciences	decidua
α-Smooth Muscle Actin (1A4)	A2547	Monoclonal	Mouse	1:4000	Sigma-Aldrich	endometrium
CD68 (Mφ) (KP1)	M0814	Monoclonal	Mouse	1:200	Dako	lymph node
CD45 (pan-leukocytes)	GA751	Monoclonal	Mouse	1:4	Dako	endometrium
CD56 (NK cells) (123C3)	M7304	Monoclonal	Mouse	1 : 50 (neonates), 1 : 100 (adult)	Dako	endometrium
SUSD2 (W5C5)	PA5-33064	Polyclonal	Rabbit	1:25	Invitrogen	neonatal lung
PDGFR-β (42G12)	LS-B3667/144,912	Monoclonal	Mouse	1:50	LifeSpan BioSciences	neonatal lung
CD90 (EPR3133)	ab133350	Monoclonal	Rabbit	1:200	abcam	endometrium
CD105 (EPR10145-12)	ab169545	Monoclonal	Rabbit	1:400	abcam	neonatal lung

#### Immunohistochemistry

The details of immunohistochemical staining procedures are described elsewhere [25, 26]. Briefly, 4 μm thick paraffin-embedded tissue sections were deparaffinized in xylene and rehydrated in graded ethanol and distilled water. Antigen retrieval was done for respective antigens. After immersion in 0.3% H_2_O_2_-methanol to block endogenous peroxidase activity (30 min), sections were pre-incubated with blocking buffer for 1 h and then incubated overnight at 4 °C with respective primary antibodies. Sections then were incubated with the secondary antibody (90 min, room temperature) followed by visualization with diaminobenzidine-H_2_O_2_. Finally, the tissue sections were counterstained with Mayer’s hematoxylin, dehydrated with serial ethanol, cleared in xylene, and mounted. A parallel staining of negative control for each slide was prepared and was incubated without primary antibody.

#### Quantification of immunoreactive cells

The immunoreactivities for ER, PGR, CD31, CD68, CD45, and CD56 in neonatal endometria were analyzed by counting the mean number of positive-staining cells in five different high power fields (HPF, x200) and were compared with positive-staining cells in adult uterus or endometrium. The cell proliferation index (Ki-67 index) in each tissue was calculated by measuring the mean percentage of Ki-67-positive nuclei among total cells in five different microscopic fields (x200). The immunoreactivity for each of EpCAM, CD10, PRL, IGFBP1, Gd-A, αSMA, SUSD2, PDFGRβ, CD90, CD105 in the samples of endometria was quantified by immunoreactive score (IRS) system as reported elsewhere [27, 28]. IRS is calculated by multiplying the staining intensity (category A) and the percentage of immunoreactive cells (category B). The staining intensity was graded as 0 (no staining), 1 (weak immunostaining), 2 (moderate immunostaining), and 3 (strong immunostaining). The percentage of immunoreactive cells was graded as 0 (0%), 1 (< 10%), 2 (10 ~ 50%), 3 (50 ~ 80%), 4 (> 80%). Multiplication of category A and B resulted in an IRS ranging from 0 to 12. We represented IRS in each endometrial sample by combined immunoreactive cells in surface epithelium, glandular epithelium and stromal compartment if not mentioned. We calculated the number of immunoreactive cells and mean IRS of five different fields of one section by light microscopy at moderate magnification (x200). Counting of all stained cells against respective markers and calculation of IRS in endometrial samples were performed by a single investigator (KO) who was blind to clinical data.

### Statistical analysis

All results are expressed as mean ± SD, mean ± SEM or median and interquartile ranges. The clinical characteristics of the subjects between groups were analyzed by one-way analysis of variance (ANOVA). Any difference in the expression of biological markers and number of immune cells between groups was analyzed by the Mann-Whitney U test. Kruskal-Wallis test was used to determine any difference among groups. Any correlation in the expression of different markers between groups was analyzed by Pearson product-moment correlation coefficient. The distribution of each marker between groups was expressed using the box and whisker plots with the medians and inter-quartile range (IQR). A value of p < 0.05 was considered statistically significant. All data analyses were conducted using SAS software version 9.4 (SAS Institute Inc. Cary, NC, USA).

## Results

### Neonatal endometrial samples collected from autopsy cases

**Fig. 1:**
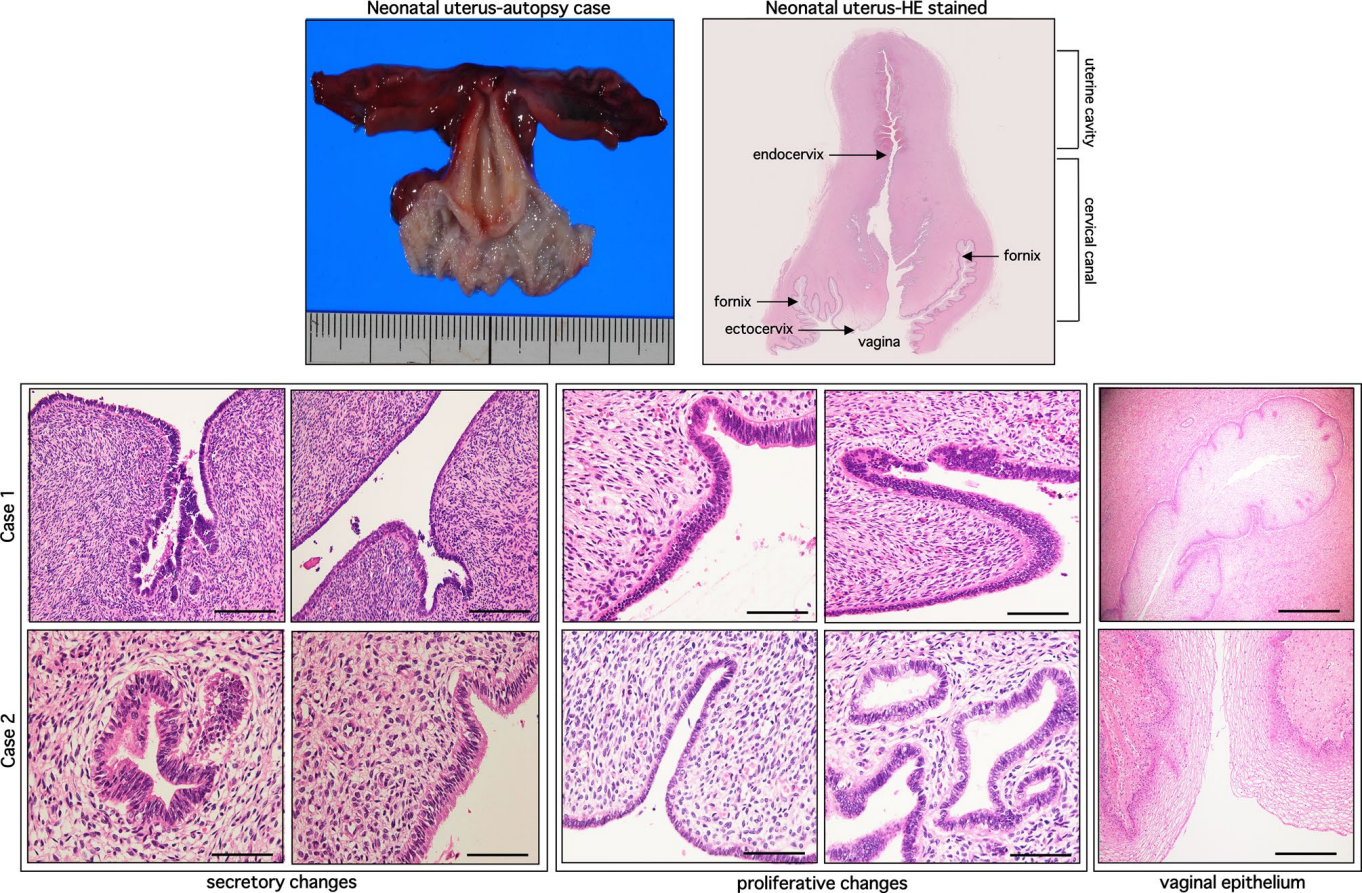
Shows images of a postmortem neonatal uterus (upper row, left), hematoxylin and eosin (HE)-stained postmortem uterus (upper row, right), and two representative cases of HE-stained neonatal endometria showing secretory and proliferative changes with autologous vaginal wall (middle and lower rows). Scale bar = 50 and 100 μm

In an attempt to understand the possible mechanistic basis in the association between NUB and early-onset endometriosis in young women, we investigated a panel of biological and eMSCs markers in neonatal endometrium and NUB of newborn babies. At postmortem examination, neonatal cervical canal was found relatively longer than the length of the uterine cavity of corpus. Among 15 cases of neonatal endometria, 13 displayed secretory change (vacuolated glands and sparse stroma) and 2 cases showed proliferative change (dense gland cells and stroma). During histological examination of the uterus collected from these postmortem cases, hemorrhage in the endometrium was found in two cases (13.4%), and a few red blood cells in uterine stroma were detected in three cases (20.0%). No hyperemia or petechial hemorrhage in endometrium was observed in any of the remaining cases. An image of a postmortem uterus and two representative cases of H&E-stained neonatal endometria showing secretory and proliferative changes with autologous vaginal wall are shown in Fig. 1. The clinical profiles of 15 autopsy cases, diagnosis at autopsy and histology of the uterus are shown in Table 2.

**Table 2:** Characteristics of 15 autopsy cases from whom neonatal endometria were collected for current study

Case No.	Gestational (wks)	age(d)	Mode of delivery	BW (g)	Hormonal dating*	Death after birth (day)	Diagnosis at autopsy	Histology of uterus
1	29	6	elective CS	1350	secretory change	17	Hydrops fetalis, pleural fluid	Hyperemia (-)/Petechiae (-)
2	38	4	elective CS	2610	secretory change	32	Pulmonary atresia, VSD	Hyperemia (-)/Petechiae (-)
3	30	1	elective CS	1428	secretory change	12	Ebstein anomaly, ELBW	Hyperemia (-)/Petechiae (-)
4	32	3	elective CS	2152	secretory change	1	Polysplenia syndrome, AVC	Hyperemia (-)/Petechiae (-)
5	38	0	normal VD	1942	secretory change	10	Ebstein anomaly	Hyperemia (-)/Petechiae (-)
6	27	5	elective CS	715	secretory change	1	Twin preterm PROM, DORV	Hyperemia (-)/Petechiae (-)
7	36	2	normal VD	2656	secretory change	18	Severe congenital ichthyosis	Hyperemia (-)/Petechiae (-)
8	34	6	normal VD	1972	secretory change	stillbirth	Thanatophoric dysplasia (dwarfism)	Hyperemia (-)/Petechiae (-)
9	38	0	elective CS	2400	secretory change	2	Congenital diaphragmatic hernia	RBCs in uterine stroma
10	39	0	selective CS	2500	secretory change	1	Congenital diaphragmatic hernia	Hemorrhage in endo
11	40	0	normal VD	3330	secretory change	0	Sudden infant death syndrome	RBCs in uterine stroma
12	28	4	elective CS	1419	secretory change	0	Congenital diaphragmatic hernia	Hyperemia (-)/Petechiae (-)
13	36	1	normal VD	2280	secretory change	0	Tetralogy of Fallot	RBCs in uterine stroma
14	37	6	selective CS	2994	proliferative change	0	CC adenomatoid malformation of lung	Hyperemia (-)/Petechiae (-)
15	38	6	normal VD	2500	proliferative change	8	Total anomalous PV connection, CHD	Hemorrhage in endo

*Hormonal dating was confirmed by hematoxylin-eosin stained slides of neonatal endometria. RBC, red blood cells;

CS, Caesarian section; VD, vaginal delivery; VSD, ventricular septal defect; ELBW, extreme low birth weight; AVC, atrio-ventricular

communis; PROM, premature rupture of membrane; DORV, double outlet RV; CC, congenital cystic, CHD, congenital heart disease

### Expression profiles of EpCAM, CD10, ER, PGR, Ki-67 and CD31 in neonatal endometria

**Fig. 2:**
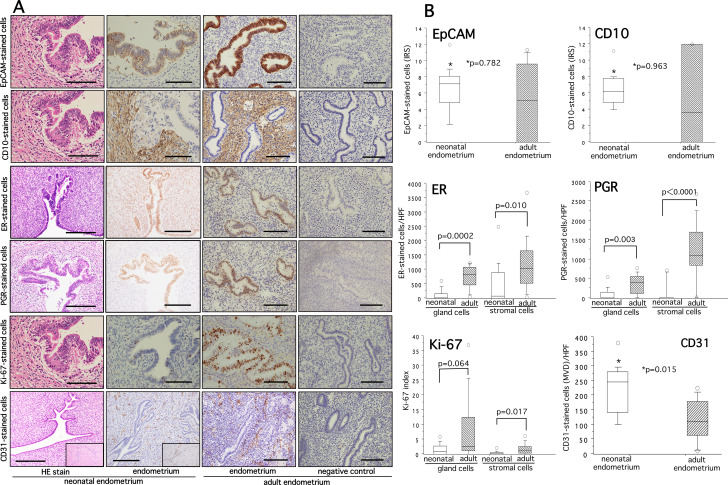
Hematoxylin and eosin staining (HE stain) and immunohistochemical staining of EpCAM, CD10, ER, PGR, Ki-67, and CD31 in neonatal and adult endometria are shown in panel (**A**). The quantitative analysis of EpCAM-, CD10-, ER-, PGR-, Ki-67-, and CD31-stained cells and significance between groups for each marker is shown on the right panel (**B**). The details of quantitative analysis of each marker are described in methods. A slide of negative controls without use of first antibody is shown on the extreme right row of panel (**A**). The boxes represent the interquartile ranges and horizontal lines in the boxes represent median values. EpCAM, marker of glandular epithelial cells; CD10, marker of stromal cells; ER, estrogen receptor; PGR, progesterone receptor; Ki-67, marker of cell proliferation; CD31, marker of vascular cells. Scale bar = 50 and 100 μm

A slide image illustrating immunoexpressions of EpCAM, CD10, ER, PGR, Ki-67, and CD31 in neonatal and adult endometria is shown in Fig. 2A. Among 15 autopsy cases, the immunoreactivity rates of all biological markers in either gland cells and/or stromal cells of neonatal endometria are shown in Suppl. Table 1. We quantified the immunoreactivity of these markers that showed a range of weak, moderate to strong staining intensity. After quantification of immunoreactivity for each marker, no significant difference was found in EpCAM- and CD10-stained cells between neonatal and adult endometria (Fig. 2B, upper panel). Both gland cells and stromal cells showed a significantly lower immunoexpressions of ER and PGR in neonatal endometria as compared with the adult endometria (ER, gland cells, P = 0.0002, stromal cells, P = 0.010; PGR, gland cells, P = 0.003, stromal cells, P = 0 < 0.0001 (Fig. 2B, middle panel). While no difference was found in Ki-67-index in glandular epithelial cells between neonatal and adult endometria (P = 0.064), a significantly decreased Ki-67 index was observed in the stromal component (P = 0.017) (Fig. 2B, lower left panel). In contrast, the micro-vessel density (MVD) as measured by CD31-stained cells was significantly higher in neonatal endometria than that in adult endometria (P = 0.015) (Fig. 2B, lower right panel). The MVD appeared to be significantly higher in neonatal endometria than in neonatal myometria (P = 0.004) (data not shown).

### Expression profiles of decidual and pre-decidual markers in neonatal endometria

We were curious to know the decidual reaction in neonatal endometria by investigating immunoexpressions of two decidual markers (prolactin and IGFBP1) and two pre-decidual markers (Gd-A and α-SMA) and their differences in expression with adult endometria.

**Fig. 3:**
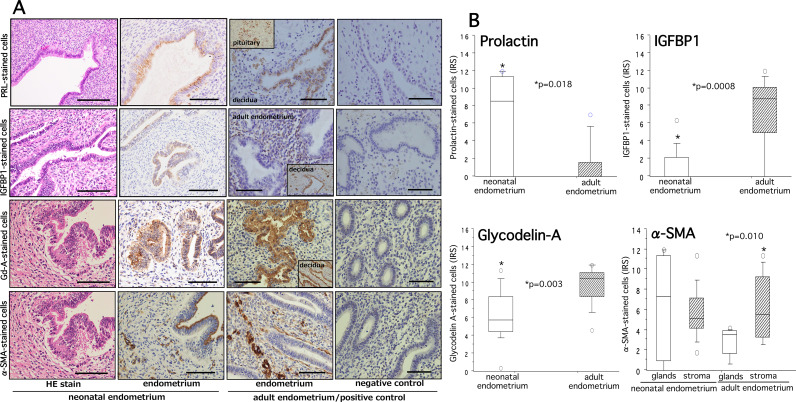
Hematoxylin and eosin staining (HE stain) and immunohistochemical staining of PRL, IGFBP1, Gd-A, and α-SMA in neonatal and adult endometria are shown in panel (**A**). The quantitative analysis of PRL-, IGFBP1-, Gd-A-, and α-SMA-stained cells and significance between groups for each marker is shown on the right panel (**B**). The details of quantitative analysis are described in methods. A slide of negative controls without use of first antibody is shown on the extreme right row of panel (**A**). In addition to adult endometria, decidual tissue and pituitary gland were used as positive controls (inset). The boxes represent the interquartile ranges and horizontal lines in the boxes represent median values. PRL, prolactin-decidual marker; IGFBP1, insulin growth factor binding protein 1- decidual marker; Gd-A, Glycodelin-A (also named placentral protein 14)-pre-decidual marker; α-SMA, alpha-smooth muscle actin-pre-decidual marker. Scale bar = 50 and 100 μm

The immunoexpression patterns of prolactin (PRL), IGFBP1, Gd-A and α-SMA in neonatal and adult endometria are shown in Fig. 3A. Ten neonatal endometria (66.7%) showed positive staining for PRL and were mainly observed in the apical surface of glandular lumen with a sparse distribution in stroma in 9 cases (60.0%). The immunoreactivity of PRL as measured by IRS in neonatal endometria was significantly higher than that in adult endometria (P = 0.018) (Fig. 3B, upper left panel). While PRL expression was found in both glandular epithelial and stromal cells, IGFBP1 expression was confined to only stromal cells of adult endometria. In quantitative analysis, the IRS of IGFBP1 in neonatal endometria was significantly lower than that in adult endometria (P = 0.0008) (Fig. 3B, upper right panel).

A strong immunoreactivity of Gd-A was found in the cytoplasm of glands cells of neonatal and adult endometria collected from all cases with a sparse weak staining in surrounding stromal cells (Fig. 3A). A moderate to strong expression of α-SMA was found in vascular/perivascular area and stromal cells of both neonatal and adult endometria with a moderate to strong staining at the apical surface of gland cells in neonatal endometria (Fig. 3A). Due to weak staining in stromal cells, we analyzed immunoreactivity of Gd-A in only gland cells and found that IRS of Gd-A was significantly lower in neonatal endometria compared with that of adult endometria (P = 0.003) (Fig. 3B, lower left panel). The IRS of α-SMA was analyzed in both gland cells and stromal compartment. While IRS of α-SMA did not show any significant difference between gland cells and stromal cells of neonatal endometria, stromal compartment of adult endometria showed a significantly higher IRS of α-SMA than that in gland cells (P = 0.010) (Fig. 3B, lower right panel).

### Expression profiles of immune cells in neonatal endometria

**Fig. 4:**
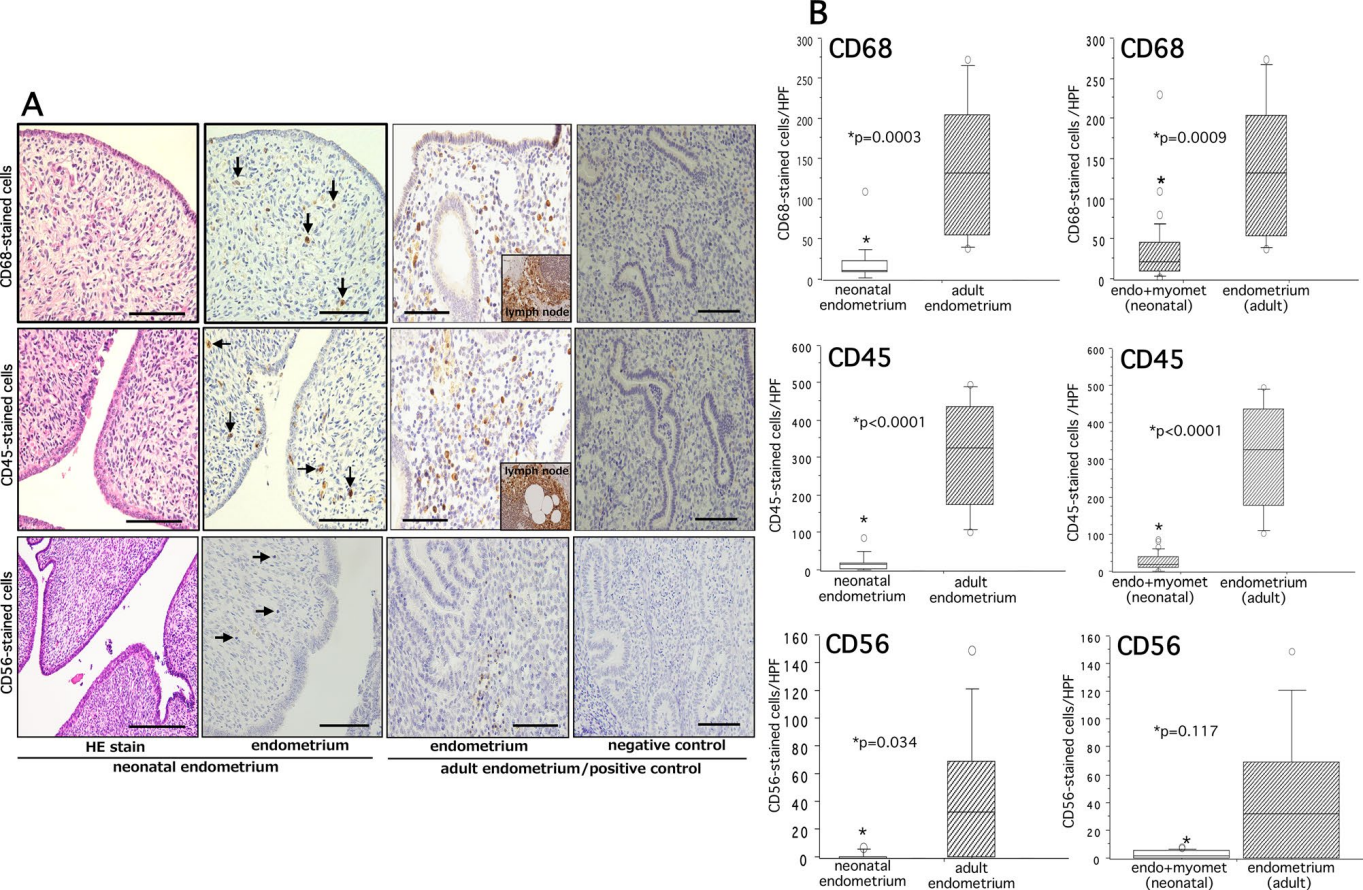
Hematoxylin and eosin staining (HE stain) and immunohistochemical staining of CD68, CD45, and CD56 in neonatal and adult endometria are shown in panel (**A**). The quantitative analysis of CD68-, CD45-, and CD56-stained cells and significance between groups for each marker are shown on the right panel (**B**). The details of quantitative analysis are described in methods. A slide of negative controls without use of first antibody is shown on the extreme right row of panel (**A**). In addition to adult endometria, lymph node tissues were used as positive controls (inset). The boxes represent the interquartile ranges and horizontal lines in the boxes represent median values. CD68, marker of macrophages; CD45, marker of pan-leukocytes; CD56, marker of natural killer cells. Scale bar = 50 and 100 μm

A slide image illustrating the CD68 (Mφ)-, CD45 (pan-leukocytes)-, and CD56 (NK cells)-immunostained cells in neonatal and adult endometria is shown in Fig. 4A. The tissue infiltrations of each of these immune cells in respective endometria and myometria were counted and expressed as the mean number of immunostained cells per HPF. We found that numbers of CD68-, CD45-, and CD56-immunostained cells in neonatal endometria were significantly less than those in adult endometria (P = 0.0003, P < 0.0001, P = 0.034, respectively (Fig. 4B, left column). Considering the thinness of neonatal endometrium, we analyzed tissue infiltration of these three immune cells in the combined endometria and myometria of neonatal uterus and compared them with those of adult endometria. We found that the numbers of CD68 + and CD45 + cells in the combined neonatal endometria and myometria were still significantly less than those in adult endometria (P = 0.0009 and P < 0.0001, respectively) without showing any significant difference for CD56 + cells (P = 0.12) (Fig. 4B, right column).

Considering that Gd-A has strong immunosuppressive effects on several human tissues and immune cells [24, 29–32], we were curious to know the tissue infiltration of different immune cells (Mφ, pan-leukocytes and NK cells) in neonatal and adult endometria and their relationship with Gd-A expressions in respective endometria.

We found that neonatal endometrial tissue showed weakly negative correlation between Gd-A expression and CD68 + cells or between Gd-A expression and CD45 + cells (Suppl. Figure 2). The correlation between Gd-A expression and CD56 + cells was lost in neonatal endometria. This negative association was not observed in adult endometria (Suppl. Figure 2). In order to demonstrate the existence of a possible immunosuppressive environment in the pelvis of newborn babies, we measured concentration of Gd-A in the peritoneal fluid (PF) of six newborn babies (n = 6) who were operated on for benign conditions and compared them with that in the neonatal uterine blood (n = 5) by ELISA. We found a small amount of Gd-A in both PF (median, 58.4 ng/mL) and NUB (median, 43.5 ng/mL) without any significant difference between them (data not shown).

### The morphological appearance and cellular contents of NUB

**Fig. 5:**
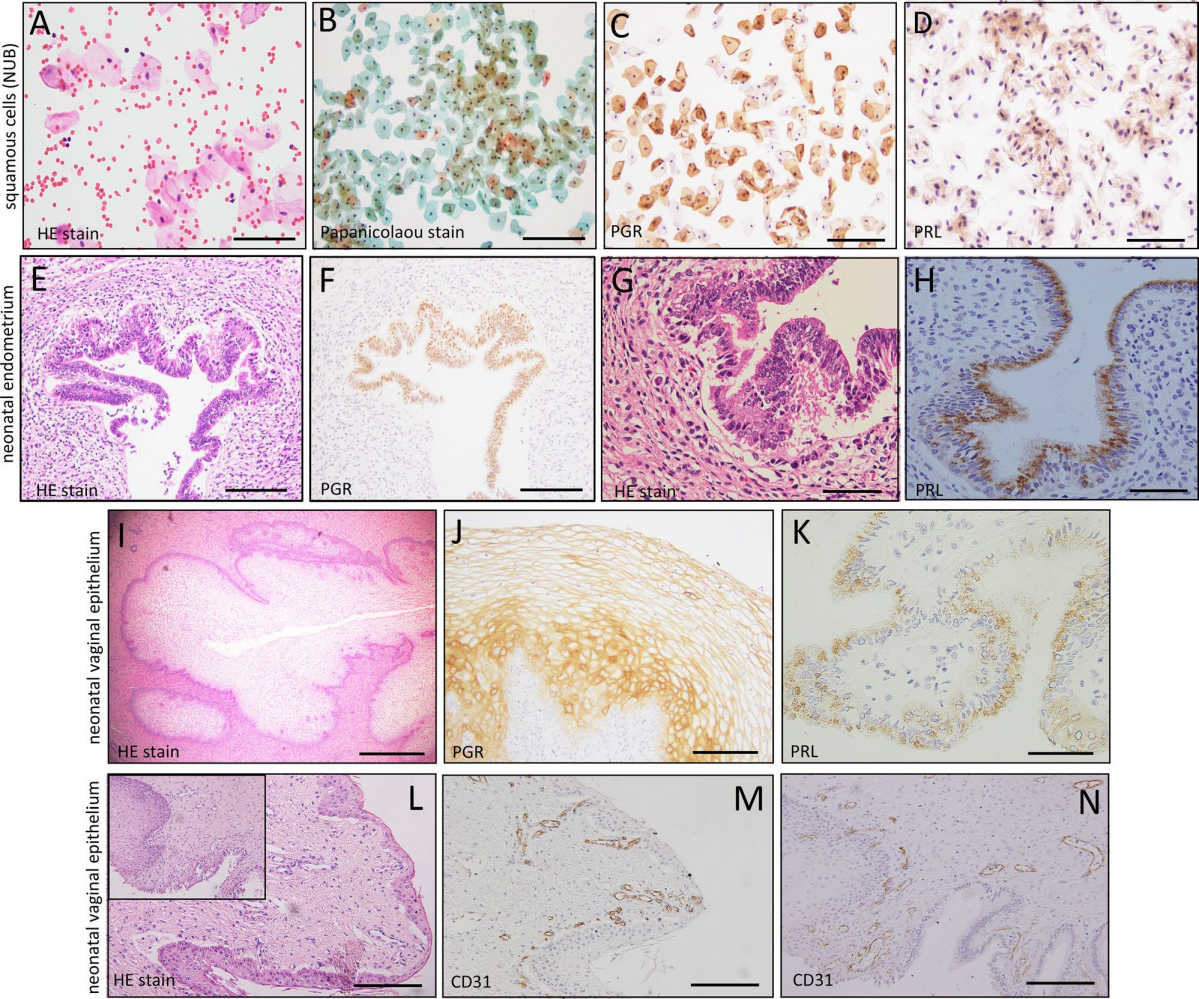
Hematoxylin and eosin staining (HE stain) (**A, E, G, I, L**), Papanicolaou (Pap) stain (**B**) and immunocytochemcial/histochemical staining of PGR (**C, F, J**), PRL (**D, H, K**), and CD31 (**M, N**) in neonatal uterine blood (NUB), neonatal endometria, and neonatal vaginal epithelium. An abundant collection of vaginal squamous cells (VSCs) in NUB and a variable positive PGR- and PRL- stained cells were observed in VSCs (**C, D**), neonatal endometria (**F, H**) and in neonatal vaginal epithelial cells (**J, K**). Both superficial and deeper part of vaginal wall shows increased number of CD31-stained micro-vessels (**M, N**). Scale bar = 50 and 100 μm

The collected NUB from 18 cases was morphologically analyzed by H&E stain or Pap stain. A large number of non-keratinized squamous cells were seen in NUB against the background of erythrocytes (Fig. 5A, B). The cytoplasm staining of most squamous cells appeared as turquoise (greenish blue color) and some of them appeared to be intermediate squamous cells (Fig. 5B). There were a few scattered leukocytes among other cells. There was no columnar epithelial cell appearing to be of endometrial origin. Following the morphological analysis, we cut the sheet and transferred the cells to several new slides by cell transfer method and performed immunocytochemistry using the respective antibodies. Squamous cells in NUB showed strong positive cytoplasmic reaction to PGR, Gd-A, and SUSD2 but weak or no staining for other markers (EpCAM, CD10, ER, PRL, IGFBP1, α-SMA) and/or other eMSCs markers (PDGFRβ, CD90, CD105) (Suppl. Figure 3). All these findings indicate the absence of any epithelial/stromal cells and/or eMSCs in NUB. Interestingly, findings of PGR- and PRL-stained squamous cells in NUB (Fig. 5C, D) were well coincided with the immunostaining of PGR and PRL in several neonatal endometria and neonatal vaginal epithelia (Fig. 5F, H, J, K). Moreover, superficial and deeper part of neonatal vaginal wall showed abundant CD31-stained micro-vessels (Fig. 5M, N). From these findings, we cannot rule out the possibility that part of the NUB may originate from the neonatal vaginal wall and coincides with the findings of previous reports [7, 11, 16].

### Expression profiles of eMSCs in neonatal endometria

**Fig. 6:**
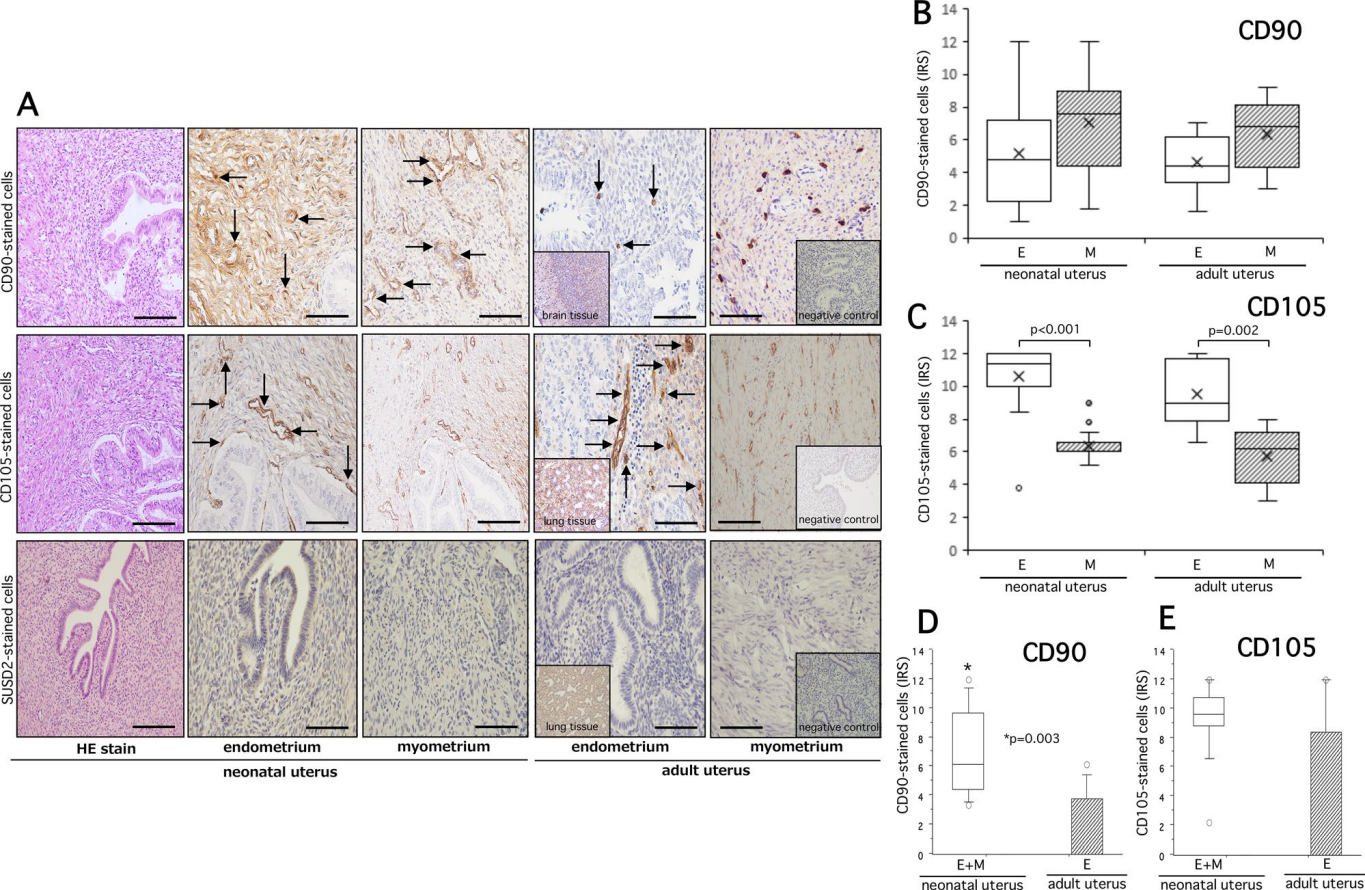
Hematoxylin and eosin staining (HE stain) and immunohistochemical staining of CD90 (upper row), CD105 (middle row), and Sushi domain containing 2 (SUSD2) (lower row) in the neonatal endometria/myometria and adult endometria/myometria of panel **A**. Arrows indicate CD90- or CD105-stained cells in respective endometria or myometria. No SUSD2-stained cells were identified in neonatal and adult endometria or myometria. In addition to adult endometria, neonatal brain tissue (inset) and lung tissue (inset) were used as positive controls for CD90, CD105, and SUSD2. Respective slides of negative controls (inset) without the use of first antibody are shown on the extreme right slides of panel (**A**). The quantitative analysis of CD90- and CD105-stained cells in endometria (E), myometria (M) and combined endometria + myometria (E + M) and significance between groups for each marker are shown on the panel (**B, C, D, E**). The details of quantitative analysis are described in methods. The boxes represent the interquartile ranges and horizontal lines in the boxes represent median values. Scale bar = 50 and 100 μm

Since Pap stain, cell block and cell transfer methods and immunocytochemical analysis could not identify any expected eMSCs in NUB, we extended further study to identify the presence of any eMSCs in neonatal endometria using the similar target markers of eMSCs. While immunohistochemical analysis could identify some CD90 + and CD105 + eMSCs in neonatal/adult endometria and myometria (Fig. 6A, upper and middle column), our analysis could not detect any SUSD2+ (Fig. 6A, lower column) and PDGFRβ + cells (data not shown) in neonatal/adult endometria and myometria. The immunoreaction of CD90- and CD105-stained cells was mostly localized to the spindle shaped pericytes of vascular wall and/or perivascular area both in endometria and myometria. Our quantification analysis revealed that IRS of CD90-stained cells did not show any significant difference between endometria (E) and myometria (M) of neonatal and adult uterus (Fig. 6B). In contrast, IRS of CD105-stained cells displayed a statistically significant increased distribution in the neonatal endometria (E) (P < 0.001) and adult endometria (E) (P = 0.002) than that in corresponding myometria (M) (Fig. 6C). Considering the thinness of the neonatal endometria, we compared CD90- and CD105-stained cells in the combined endometria + myometria (E + M) of neonatal uteri with that in adult endometria. We found a significantly higher distribution of CD90-stained cells in the neonatal E + M compared with that in the adult endometria (P = 0.003) (Fig. 6D). This difference was lost for CD105-immunoreactive cells (Fig. 6E).

## Discussion

In this study, we investigated a long overlooked issue regarding the involvement of NUB in the development of early-onset endometriosis. Based on our serial experimental evidences, we did not find any supporting evidence for the role of NUB in early-onset endometriosis. While our data suggest that neonatal endometrial cells may have some potential in early-onset endometriosis, it is unclear as this moment as how they could be transported and deposited into the peritoneal cavity, seemingly resistant to removal, and stay in the cavity for years and then at the onset of thelarche become activated and form endometriotic lesions.

It is hypothesized that there is a different mechanism in early-onset endometriosis in pre-menarcheal girls [33]. The presence of eMSCs in NUB has been thought to be the origin of early-onset endometriosis [8]. In our current study, however, we could not detect either of endometrial epithelial/stromal cells or eMSCs cells in NUB, even though we did detect markers of eMSCs in neonatal endometrium. More surprisingly, we found abundant number of squamous cells of vaginal origin, instead of endometrial epithelial/stromal cells, in NUB. In our separate experiments with postmortem neonatal endometria, we found that similar to adult endometria, neonatal endometria expressed EpCAM-positive and CD10-positive cells, exhibited decidual reaction, and displayed a substantial amount of proliferative and angiogenic activity. Unlike NUB, we could detect some phenotypes of eMSCs such as CD90 + and CD105 + cells in neonatal endometria. Therefore, with these data, we found no evidence in support for the hypothesis that NUB contains eMSCs, which are the putative seeds responsible for early-onset endometriosis [8, 12–14].

NUB is considered to be the withdrawal bleeding due to a sudden decrease of placental progesterone after parturition, a physiological phenomenon similar to adult menstruation. The fetal or neonatal endometrium is influenced by the maternal circulating hormones rather than by the own ovaries. It is controversial whether NUB comes from endometrium or from cervix but the widely accepted theory is that it originates from the endometrium. In this study, the ER and PGR expression was seen in 50% and 15%, respectively, of neonatal endometria and the PGR expression was increased at term. The neonatal endometria showed positive reaction to ER and PGR, although the expression in terms of intensity was significantly lower than that of adults. There was also a single layer of endometrial epithelium and morphologically showed the secretory change in most cases, consistent with the previous report [17].

Of note is the apparent discrepancy in ER staining between NUB and neonatal endometria, highlighting our observation that NUB is highly likely to originate from vaginal epithelial cells, not from endometrium as previously thought. As such, the NUB hypothesis may be in need of a complete overhaul.

Decidualization is a reaction that occurs around vessels in the stroma under the influence of progesterone. In this study, PRL, one of the decidual markers, was significantly higher in neonatal endometria than in adult endometria. As for IGFBP, another decidual marker, was significantly higher in adult endometria than in neonatal endometria. These findings seem to suggest that decidualization in endometria may differ between adults and neonates.

One of the interesting findings of our study is that tissue infiltration of three different immunocompetent cells was significantly lower in neonatal endometria than that in adult endometria. These findings echo to a previous report that the distribution of immunocompetent cells, such as CD68 + macrophages, CD45 + leukocytes, and CD56 + natural killer (NK) cells in neonatal endometria differs from that in adult endometria [18]. This decreased accumulation of immune cells showed an apparent association with the strong expression of Glycodelin A (Gd-A) in neonatal endometria. It has been reported that in addition to behave as a pre-decidual marker, Gd-A exhibits strong immunosuppressive action in different human tissues and immune cells such as monocytes, T-cells, and NK cells [21–24, 31]. In fact, Gd-A expression was observed in almost all of the neonatal endometria. This finding in neonatal endometria may possibly suggest the pro-survival propensity of ER + and PGR + cells should they gain the entry into the pelvis. However, how they can be transported, presumably in a piecemeal fashion, into the peritoneal cavity remains unclear. This is of particularly interest since the window of NUB is in a matter of days, practically a very short time window as compared with many opportunities of retrograde menstruation in adult women. Moreover, even if they gain the entry to the pelvic cavity, how they can gain supply of nutrients, macromolecules, and oxygens, and thus secure their long-term survival remains completely unknown. Therefore, our findings, in conjunction of these considerations, suggest that while NUB might be responsible for early-onset endometriosis, much more data are needed to establish this causal link.

Regulatory T (Treg) cells are potent suppressors of inflammatory immune responses and are essential in preventing destructive immunity in all tissues thereby protecting our body against infection or development of a lesion [34, 35]. A delicate balance between pro-inflammatory molecule (Th17 cells) and anti-inflammatory molecule (Treg cells) operates in pelvic environment and may be involved in the onset of early endometriosis as recently reported in adult women [36]. Manipulation of NK and NK T cells (NKT) is considered to be a useful tool for immunotherapy in endometriosis and cancer [37–39]. As one of the immunosuppressive molecules, our knowledge on Gd-A in neonatal endometria is insufficient and scarcely described. Traditionally, Gd-A, known previously as placental protein 14 (PP14), a secreted immunosuppressive glycoprotein, has been considered as an indispensable macromolecule in the maternal system for the establishment, maintenance, and progression of pregnancy [40, 41]. Down-regulation of glycodelin leads to increased activation of the maternal immune system and can result in abortion during the first trimester [42].

Gd-A suppresses the function of T cells and/or NK cells by its direct anti-proliferative and/or pro-apoptotic action on these immune cells [29–31, 40, 41]. It has been reported that endometriosis patients had significantly higher serum and peritoneal fluid (PF) concentrations of Gd-A compared with control women without endometriosis and displayed Gd-A expression in endometriosis lesions [43, 44]. All these findings indicate that Gd-A exhibits a potential role in creating an immunosuppressive environment in pelvis and in the pathogenesis of endometriosis. Based on decreased number of immune cells in neonatal endometria in our current study, we can postulate that a decreased immune environment in neonates may prevent eMSCs and/or endometrial cells from being eliminated by immune cells if they flow back to the pelvis. The significantly less distribution of immune cells in neonatal endometria as an alternative mechanistic basis of early-onset endometriosis may well coincide with the accepted hypothesis of endometriosis that a defective immune response contributes to the implantation of retrograde endometrial cells in pelvis during menstruation [45].

Endometrial stem/progenitor cells were found in the menstrual blood and PF of women with and without endometriosis [46]. It is expected that eMSCs in menstrual blood retrogrades into pelvis and may play a role in the pathogenesis and progression of endometriosis. However, eMSCs have not been identified in NUB in our study. As an observation study with small samples, we could not detect any eMSCs in PF of newborn babies (Ogawa et al., unpublished data). SUSD2, CD146, PDFGRβ are the markers used for separating eMSCs by FACS analysis [46]. CD146, CD90, CD105 positive cells were found in perivascular stroma in adult endometria [10]. We demonstrated that CD90 + and CD105 + cells are similarly located in the perivascular area in neonatal endometria, while we could not find any CD90 + or CD105 + cells in NUB by our current methodology. Even CD90 + and CD105 + eMSCs cells in neonatal endometria remain dormant in pelvis until puberty after their retrograde transport, these cells lack ER and PGR [9, 10]. In the absence of steroid hormonal receptors, it is therefore difficult to understand that eMSCs in NUB and/or in neonatal endometria can differentiate into endometriosis-like tissues or cells under the influence of ovarian steroids during puberty. Therefore, the role of eMSCs in NUB and/or in neonatal endometria in early-onset endometriosis still remains to be an equivocal issue yet to be confirmed by future study.

It is also unclear as why the presumably dormant endometrial cells and/or eMSCs, if any, in pelvis are not removed by the immunocompetent cells until puberty. Our current findings of decreased immunocompetent cells and their association with strong expression of Gd-A in neonatal endometria may, at least in part, clarify this issue. Although a proportion of niche cells among eMSCs do express ER and PGR [10], their existence and viability in NUB and in neonatal endometria is unknown and yet to be determined. However, we cannot exclude the possibility that retrograde flow of ER+/PGR + cells as niche cells may affect the growth of eMSCs, because it has been reported that proliferation of some phenotypes of eMSCs is induced by the niche cells in cell co-culture system [47, 48]. Future application of different methodological tool may address this unclear issue. Alternatively, it is possible, at least theoretically, that the proportion of eMSCs is genuinely low, hence we could not detect them. Indeed, if the proportion of eMSCs + NUB is 1%, then we would have a very high probability (83.5%) of seeing no eMSCs in our 18 samples. Hence our data ruled out the possibility that a high proportion of NUB samples contain eMSCs.

We collected NUB by a non-invasive way in which blood was gently collected from diapers or vagina. NUB contains numerous squamous cells but no endometrial cells or eMSCs. Intrauterine hemorrhage and decidual change in female babies occur [17]. We also demonstrated that hemorrhage was seen in some cases of neonatal endometria. In addition to overt NUB, occult NUB occurs in 21–61% of female neonates [7, 8]. The relatively long neonatal cervix as compared with adult one may block the cervix by mucus plug. These features of neonatal uterus may facilitate the regurgitation of NUB. Accordingly, “no visible NUB” does not mean that “no bleeding in endometrium”. At this moment, overt NUB cannot be a reliable biomarker of future endometriosis. On the other hand, the pattern of expression of higher PGR and PRL expression in neonatal vaginal wall with increased vascularity is suggestive of the possibility that part of the NUB could originate from the vaginal wall. Similar to the effect on neonatal endometria, surface vaginal mucosal tissue may become fragile and cause bleeding that is enriched with vaginal squamous cells as a result of maternal P withdrawal during parturition. In fact, uterine bleeding in the neonate was first described as vaginal bleeding by Carus in 1822 and later by Drake in 1907 [11] and may support our current findings. Further studies are indeed necessary to clarify this issue.

This study has several strengths. First, the combined use of NUB samples and the neonatal endometrial tissue samples was performed. Second, we analyzed both eMSCs and endometrial cells in NUB and neonatal endometrial samples. Third, we analyzed different immunocompetent cells and an immunosuppressive molecule (Gd-A) in neonatal endometrial tissue samples. There are some potential limitations in our current study: (1) Sample size of NUB and postmortem neonatal endometria is rather small; (2) The sample volume of NUB was scant to confirm the presence of endometrial stem/progenitor cells and/or endometrial epithelial/stromal cells by mRNA expression or FACS assay; (3) We could not clarify one important issue as whether NUB truly enters into the pelvis of female babies via the Fallopian tubes. Our unpublished data with PF (n = 6) showed presence of occult blood in a proportion of PF using H&E and Pap stain. This finding should be interpreted with caution. The presence of blood in PF could be the result of retrograde flow of NUB or contamination during surgical manipulation. (4) We did not investigate occult NUB nor did we clarify the association between non-NUB cases resulting from P resistance and occurrence of pregnancy-related complications as described before [8, 11]. In fact, this was not the focus of our current study. Future studies, with larger sample size and improved methodological tools, are warranted to address these unclear issues.

## Conclusions

Our current findings may have some biological implication to better understand the biology and pathophysiology of neonatal endometrium. Our serial experimental findings suggest that while neonatal endometria contain eMSCs and Gd-A, no eMSCs or even endometrial cells were found in NUB. Therefore, at this moment, NUB hypothesis is not supported by our current data. The origin of NUB should be carefully considered, whether it is from endometrium or from vaginal wall. As an alternative mechanism, decreased infiltration of immunocompetent cells and increased expression of Gd-A may support the possibility that neonatal endometrial cells may remain dormant in pelvis for years until puberty without being removed by immune cells. Although it is premature to conclude unequivocally as of now, we can, at least, suggest that some ER + and PGR + residual cells of neonatal endometria can be reactivated with the onset of thelarche and may culminate in the development of early-onset endometriosis. Our current study may open new avenue for future NUB study with large sample size and application of modified technological tools to confirm the existence of any eMSCs and/or endometrial cells in NUB and to clarify their biological or clinical significance.

## Electronic supplementary material

Below is the link to the electronic supplementary material.

Supplementary Material 1: **Suppl. Figure 1** Shows cell transfer methods. A representative procedure of neonatal uterine blood (NUB) collection (**A, B, C**) and steps of cell transfer method (**D-H**) are shown in this figure. The details of NUB collection and cell transfer procedure are mentioned in method section.

Supplementary Material 2: **Suppl. Figure 2**. Correlation between immunoreactive scores (IRS) of Glycodelin-A (Gd-A) expression and tissue infiltration of CD68 (macrophages)-, CD45 (pan-leukocytes)-, and CD56 (natural killer cells, NK)-stained cells (mean numbers per high power field) in neonatal endometria (**A**) and adult endometria (**B**). A weakly negative correlation between IRS of Gd-A expression and tissue infiltration of CD68-stained macrophages (r = 0.01) and CD45-stained pan-leukocytes (r = 0.22) was found in neonatal endometria but not between IRS of Gd-A expression and CD56-stained NK cells (r = 0.31) (**A**). A weakly positive correlation was observed between IRS of Gd-A expression and CD68/CD45-stained cells in adult endometria (**B**).

Supplementary Material 3: **Suppl. Figure 3** Shows expression of different biological markers in neonatal uterine blood. Immunocytochemical staining of different biological markers (**A-H**) and epithelial mesenchymal stem cells (eMSCs) (**I-L**) in prospectively collected neonatal uterine blood (NUB). Squamous cells in NUB showed strong positive cytoplasmic reaction to PGR (**D**), Gd-A (**G**), and SUSD2 (**I**) and weak or no staining for other markers (EpCAM, CD10, ER, PRL, IGFBP1, α-SMA) and/or other eMSCs markers (PDGFRβ, CD90, CD105). The respective abbreviation for each of these biological markers is mentioned in the text.

Supplementary Material 4: **Suppl. Table 1**. Shows immunohistochemical staining rates of different biological markers in neonatal endometria.

## Data Availability

The data underlying this article will be shared on reasonable request to the corresponding author.

## References

[1] Giudice LC, Kao LC (2004). Endometr Lancet.

[2] Attar E, Bulun S (2006). Aromatase and other steroidogenic genes in endometriosis: translational aspect. Hum Reprod Update.

[3] Zondervan KT, Becker CM, Missmer SA, Endometriosis (2020). N Engl J Med.

[4] Cousins FL, O DF, Gargett CE (2018). Endometrial stem/progenitor cells and their role in the pathogenesis of endometriosis. Best Pract Res Clin Obstet Gynaecol.

[5] Taylor HS, Kotlyar AM, Flores VA (2021). Endometriosis is a chronic systemic disease: clinical challenges and novel innovations. Lancet.

[6] Brosens I, Benagiano G (2013). Is neonatal uterine bleeding involved in the pathogenesis of endometriosis as a source of stem cells?. Fertil Steril.

[7] Brosens I, Brosens J, Benagiano G (2013). Neonatal uterine bleeding as antecedent of pelvic endometriosis. Hum Reprod.

[8] Brosens I, Gargett CE, Guo SW, Puttemans P, Gordts S, Brosens JJ, Benagiano G (2016). Origins and Progression of adolescent endometriosis. Reprod Sci.

[9] Gargett CE, Schwab KE, Brosens JJ, Puttemans P, Benagiano G, Brosens I (2014). Potential role of endometrial stem/progenitor cells in the pathogenesis of early-onset endometriosis. Mol Hum Reprod.

[10] Gargett CE, Schwab KE, Deane JA (2016). Endometrial stem/progenitor cells: the first 10 years. Hum Reprod Update.

[11] Brosens I, Benagiano G (2016). Clinical significance of neonatal menstruation. Eur J Obstet Gynecol Reprod Biol.

[12] Bianchi P, Benagiano G, Brosens I (2017). Promoting awareness of neonatal menstruation. Gynecol Endocrinol.

[13] Puttemans P, Benagiano G, Gargett C, Romero R, Guo SW, Brosens I (2017). Neonatal uterine bleeding as a biomarker for reproductive disorders during adolescence: a worldwide call for systematic registration by nurse midwife. J Matern Fetal Neonatal Med.

[14] Romero R (2020). Giants in Obstetrics and Gynecology Series: a profile of Ivo Brosens, MD, PhD, FRCOG (ae). Am J Obstet Gynecol.

[15] Arcellana RC, Robinson TW, Tyson RW, Joyce MR (1996). Neonatal fellowship. McKusick-Kaufman syndrome with legal complications of hydrometrocolpos and congenital endometriosis. J Perinatol.

[16] Dekker J, Hooijer I, Ket JCF, Vejnović A, Benagiano G, Brosens I, Mijatovic V (2021). Neonatal uterine bleedings: an ignored sign but a possible cause of early-onset endometriosis - A systematic review. Biomed Hub.

[17] Ober WB, Bernstein J (1955). Observations on the endometrium and ovary in the newborn. Pediatrics.

[18] Kammarer U, Rieger L, Kapp M, Dietl, Ruck P (2003). Immunocompetent cells in the endometrium of fetusus and children. Hum Reprod.

[19] Sharma S (2014). Natural cells and regulatory T cells in early pregnancy loss. Int J Dev Biol.

[20] Lyzikova YA, Zinovkin DA, Pranjal MSI (2020). Increase in FoxP3, CD56 immune cells and decrease in glands PGRMC1 expression in the endometrium are associated with recurrent miscarriages. Eur J Obstet Gynecol Reprod Biol.

[21] Fazleabas AT, Donnelly KM, Hild-Petito S, Hausermann HM, Verhage HG (1997). Secretory proteins of the baboon (Papio anubis) endometrium: regulation during the menstrual cycle and early pregnancy. Hum Reprod Update.

[22] Bolton AE, Pockley AG, Clough KJ, Mowles EA, Stoker RJ, Westwood OM, Chapman MG (1987). Identification of placental protein 14 as an immunosuppressive factor in human reproduction. Lancet.

[23] Alok A, Mukhopadhyay D, Karande AA (2009). Glycodelin A, an immunomodulatory protein in the endometrium, inhibits proliferation and induces apoptosis in monocytic cells. Int J Biochem Cell Biol.

[24] SchneiderMA, Granzow M, Warth A, Schnabel PA, Thomas M, Herth FJF, Dienemann H, Muley T, Meister M (2015). Glycodelin: a new biomarker with immunomodulatory functions in non-small cell lung cencer. Clin Cancer Res.

[25] Khan KN, Fujishita A, Ogawa K, Koshiba A, Mori T, Itoh K, Nakashima M, Kitawaki J (2022). Occurrence of chronic endometritis in different types of human adenomyosis. Reprod Med Biol.

[26] Khan KN, Fujishita A, Suematsu T, Ogawa K, Koshiba A, Mori T, Itoh K, Teramukai S, Matsuda K, Nakashima M (2021). An axonemal alteration in apical endometria of human adenomyosis. Hum Reprod.

[27] Flores VA, Vanhie A, Dang T, Taylor HS (2018). Progesterone receptor Status predicts response to Progestin Therapy in Endometriosis. J Clin Endocrinol Metab.

[28] Pluchino N, Mamillapalli R, Wenger JM, Ramyead L, Drakopoulos P, Tille JC, Taylor HS (2020). Estrogen receptor-α immunoreactivity predicts symptom severity and pain recurrence in deep endometriosis. Fertil Steril.

[29] Kämäräinen M, Leivo I, Koistinen R, Julkunen M, Karvonen U, Rutanen EM, Seppälä M (1996). Normal human ovary and ovarian tumors express glycodelin, a glycoprotein with immunosuppressive and contraceptive properties. Am J Pathol.

[30] Okamoto N, Uchida A, Takakura K, Kariya Y, Kanzaki H, Riittinen L, Koistinen R, Seppälä M, Mori T (1991). Suppression by human placental protein 14 of natural killer cell activity. Am J Reprod Immunol.

[31] Alok A, Mukhopadhyay D, Karande AA (2009). Glycodelin A, an immunomodulatory protein in the endometrium, inhibits proliferation and induces apoptosis in monocytic cells. Int J Biochem Cell Biol.

[32] Mylonas I, Jeschke U, Kunert-Keil C, Shabani N, Dian D, Bauerfeind I, Kuhn C, Kupka MS, Friese K (2006). Glycodelin A is expressed differentially in normal human endometrial tissue throughout the menstrual cycle as assessed by immunohistochemistry and in situ hybridization. Fertil Steril.

[33] Benagiano G, Guo SW (2022). Age-depdendent phenotypes of ovarian endometriomas. Reprod Med Biol.

[34] Bettelli E, Carrier Y, Gao W, Korn T, Storm TB, Oukka M, Weiner HL, Kuchroo VK (2006). Reciprocal development pathways for the generation of pathogenic effector Th17 and regulatory T cells. Nature.

[35] Guerin LR, Prins JR, Robertson SA (2009). Regulatory t-cells and immune tolerance in pregnancy: a new target for infertility treatment?. Hum Reprod Update.

[36] Khan KN, Yamamoto K, Fujishita A, Muto H, Koshiba A, Kuroboshi H, Saito S, Teramukai S, Nakashima M, Kitawaki J (2019). Differential levels of regulatory T-cells and T-helper-17 cells in women with early and advanced endometriosis. J Clin Endocrinol Metab.

[37] Thiruchelvam U, Wingfield M, O’Farrelly C (2015). Natural killer cells: key players in endometriosis. Am J Reprod Immunol.

[38] Terme M, Ullrich E, Delahaye NF, Chaput N, Zitvogel L (2008). Natural kiler cell-directed therapies: moving from unexpected results to successful strategies. Nat Immunol.

[39] Motohashi S, Ishikawa A, Ishikawa E, Otsuji M, Iizasa T, Hanaoka H, Shimizu N, Horiguchi S, Okamoto Y, Fujii S, Taniguchi M, Fujisawa T, Nakayama T (2006). A phase I study in vitro expanded natural killer T cells in patients with advanced and recurrent non-small cell lung cancer. Clin Cancer Res.

[40] Mukhopadhyay D, Sundereshan S, Rao C, Karande AA (2001). Placental protein 14 induces apoptosis in T cells but not in monocytes. J Biol Chem.

[41] Huhtala ML, Seppälä M, Närvänen A, Palomäki P, Julkunen M, Bohn H (1987). Amino acid sequence homology between human placental protein 14 and beta-lactoglobulins from various species. Endocrinology.

[42] Toth B, Roth K, Kunert-Keil C, Scholz C, Mylonas I (2008). Glycodelin protein and mRNA is downregulated in human first trimester abortion and partillay upregulated in mole pregnancy. J Histochem Cytochem.

[43] Kocbek V, Vouk K, Mueller MD, Rižner TL, Bersinger NA (2013). Elevated glycodelin-A concentrations in serum and peritoneal fluid of women with ovarian endometriosis. Gynecol Endocrinol.

[44] Wang P, Zhu L, Zhang X (2013). The role of placental protein 14 in the pathogenesis of endometriosis. Reprod Sci.

[45] Sampson JA (1927). Peritoneal endometriosis due to menstrual dissemination of endometrial tissue into the peritoneal cavity. Am J Obstet Gynecol.

[46] Masuda H, Schwab KE, Filby CE, Tan CSC, Tsaltas J, Weston GC, Gargett CE (2021). Endometrial stem/progenitor cells in menstrual blood and peritoneal fluid of women with and without endometriosis. Reprod Biomed Online.

[47] Barragan F, Irwin JC, Balayan S, Erikson DW, Chen JC, Houshdaran S, Piltonen TT, Spitzer TL, George A, Rabban JT (2016). Human endometrial fibroblasts derived from mesenchymal progenitors inherit Progesterone Resistance and acquire an inflammatory phenotype in the Endometrial Niche in Endometriosis. Biol Reprod.

[48] Chen P, Mamillapalli R, Habata S, Taylor HS.Endometriosis Cell Proliferation Induced by Bone Marrow Mesenchymal Stem Cells. Reprod Sci. 2021;28:426–34. 10.1007/s43032-020-00294-4 32812213

