# Supplementary figures and images for: Is neonatal uterine bleeding responsible for early-onset endometriosis?
Source: Reprod Biol Endocrinol. 2023 Jun 19;21:56. doi: 10.1186/s12958-023-01099-1 (PMC10278367; doi:10.1186/s12958-023-01099-1)

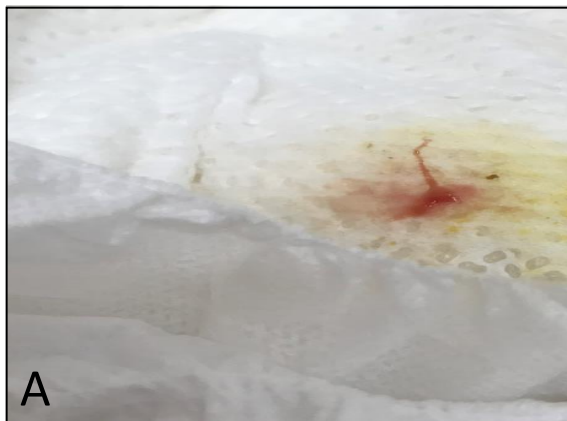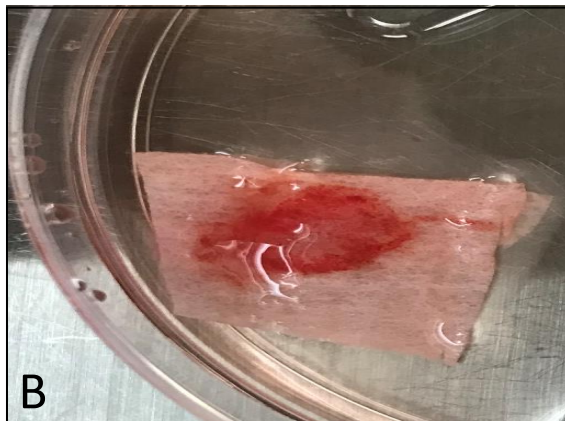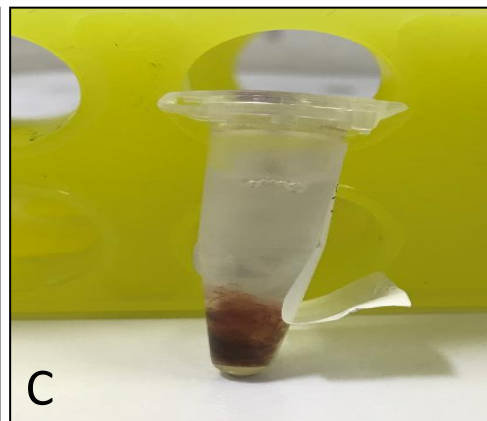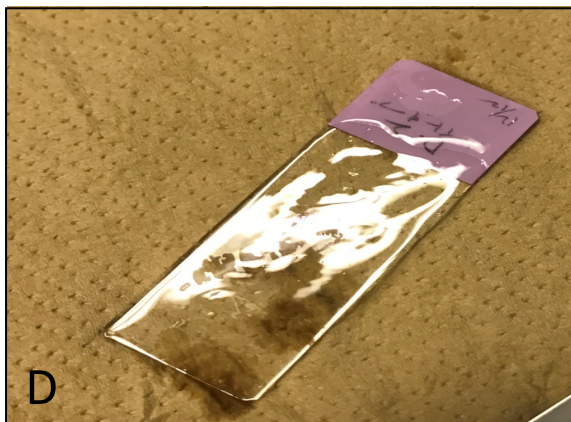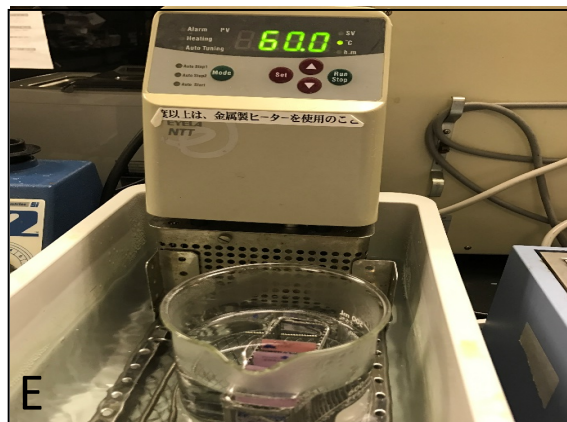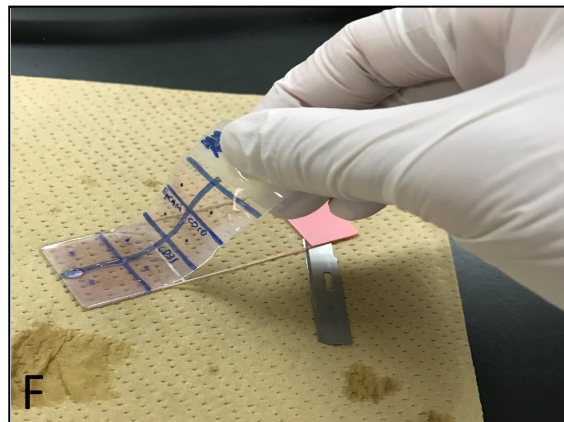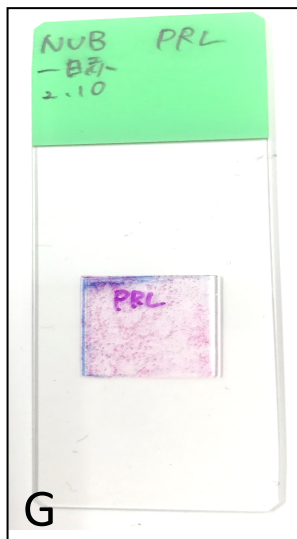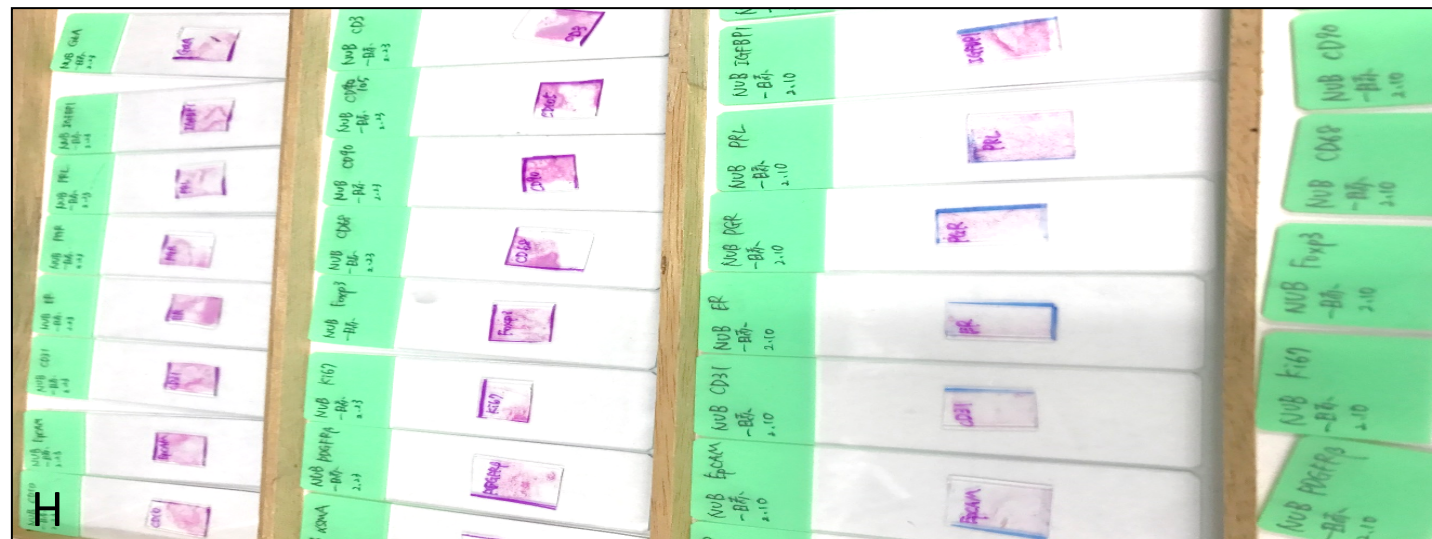

Supplement: Supplementary file 1 — Supplementary Material 1: Suppl. Figure 1 Shows cell transfer methods. A representative procedure of neonatal uterine blood (NUB) collection (A, B, C) and steps of cell transfer method (D-H) are shown in this figure. The details of NUB collection and cell transfer procedure are mentioned in method section. [file 12958_2023_1099_MOESM1_ESM.pdf]

**A**

neonatal endometrium

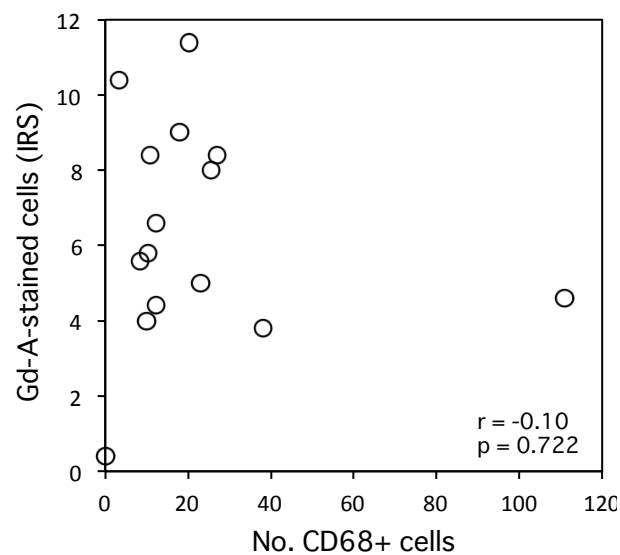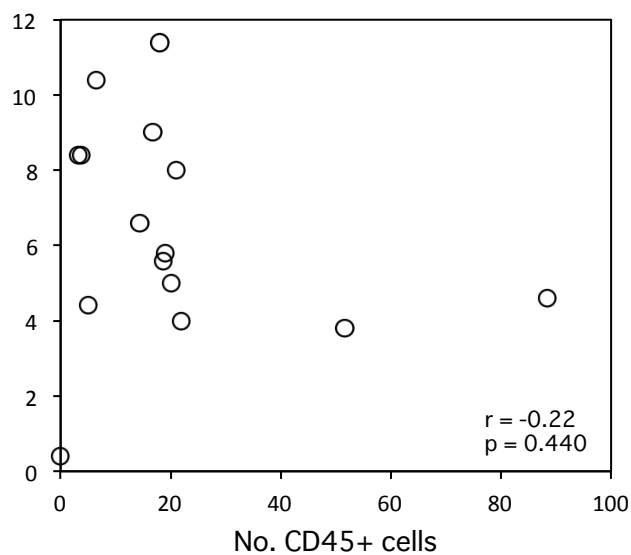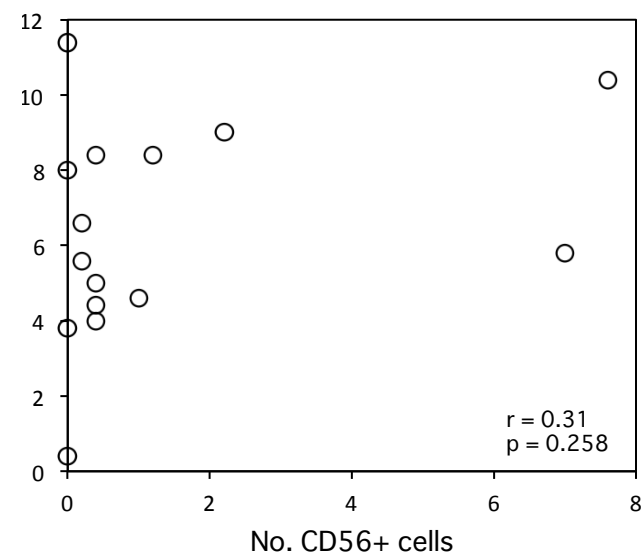**B**

adult endometrium

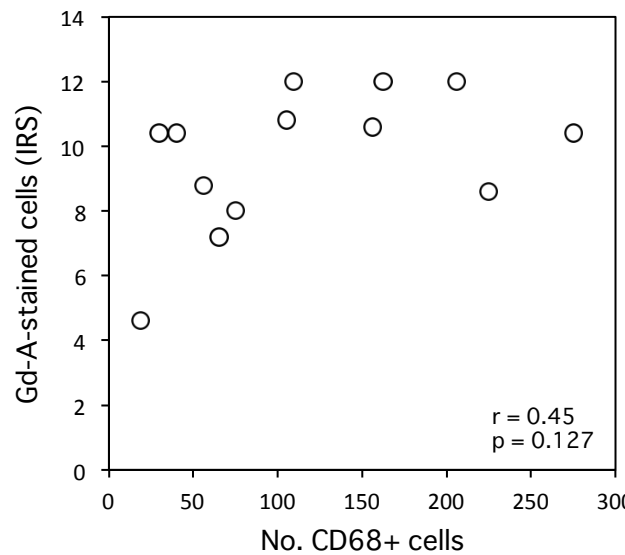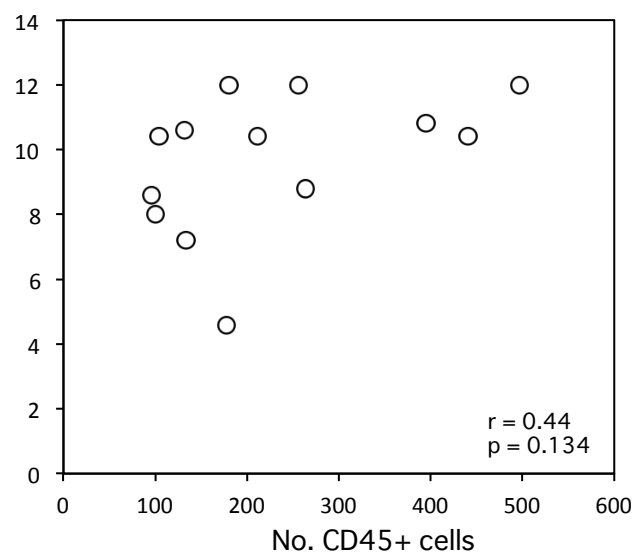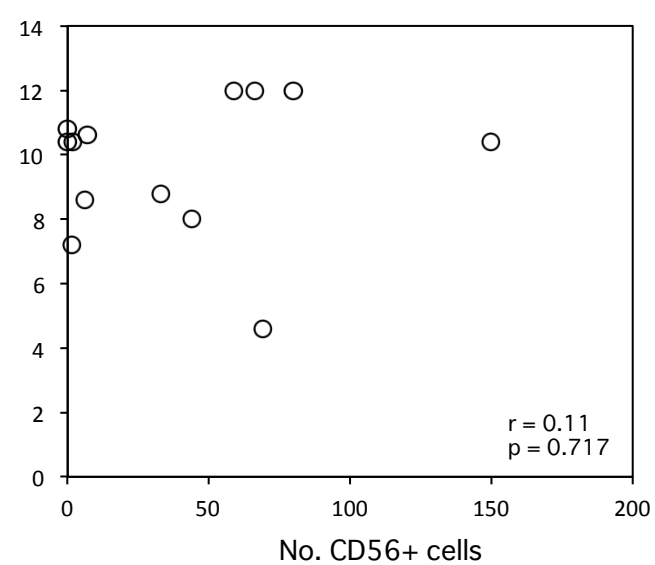

Supplement: Supplementary file 2 — Supplementary Material 2: Suppl. Figure 2. Correlation between immunoreactive scores (IRS) of Glycodelin-A (Gd-A) expression and tissue infiltration of CD68 (macrophages)-, CD45 (pan-leukocytes)-, and CD56 (natural killer cells, NK)-stained cells (mean numbers per high power field) in neonatal endometria (A) and adult endometria (B). A weakly negative correlation between IRS of Gd-A expression and tissue infiltration of CD68-stained macrophages (r = 0.01) and CD45-stained pan-leukocytes (r = 0.22) was found in neonatal endometria but not between IRS of Gd-A expression and CD56-stained NK cells (r = 0.31) (A). A weakly positive correlation was observed between IRS of Gd-A expression and CD68/CD45-stained cells in adult endometria (B). [file 12958_2023_1099_MOESM2_ESM.pdf]

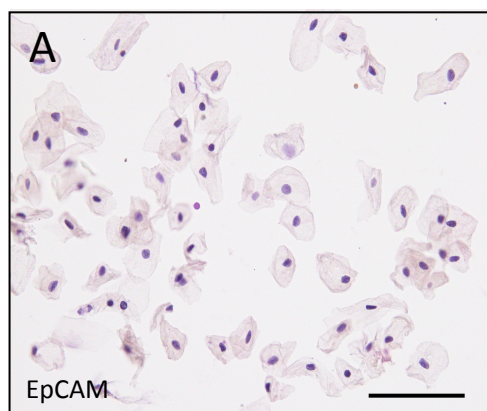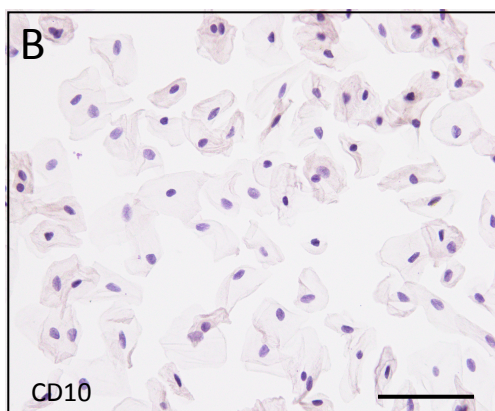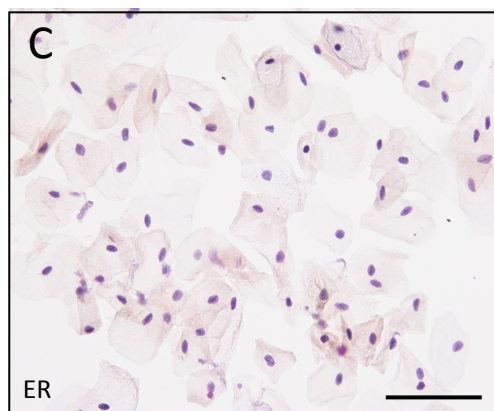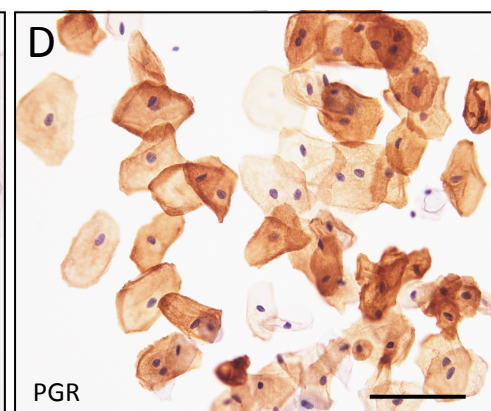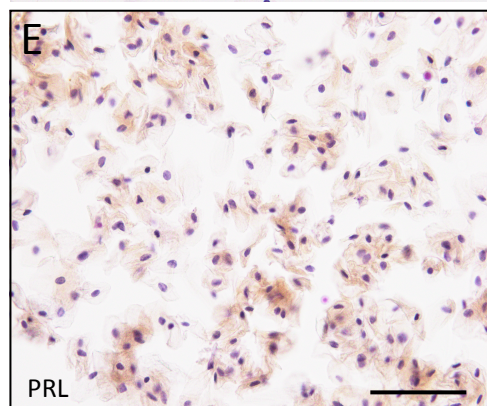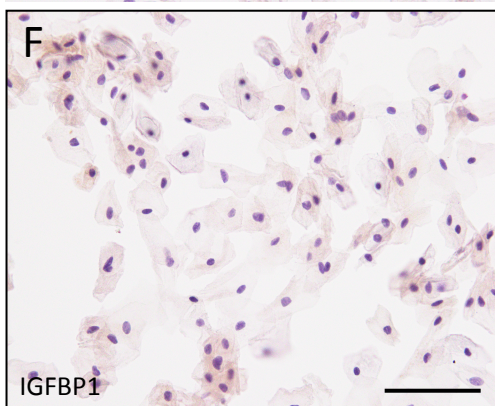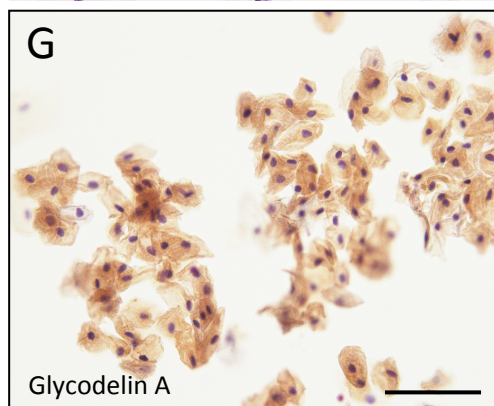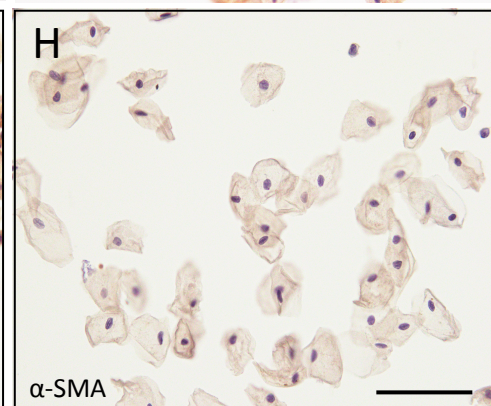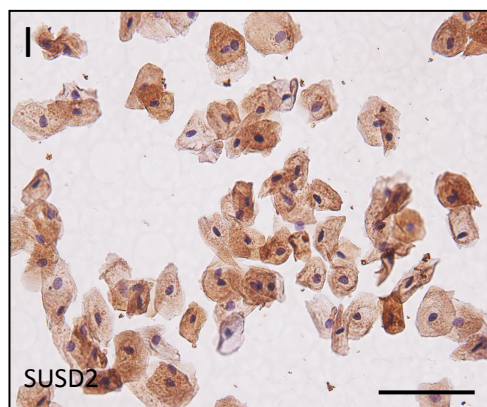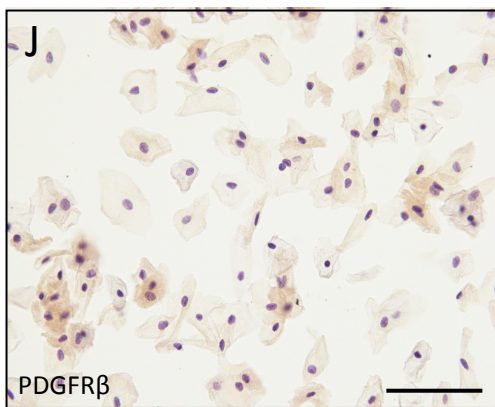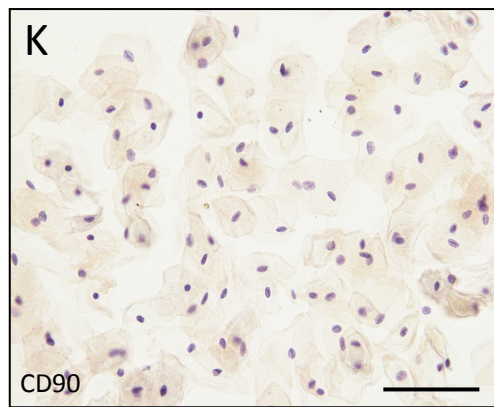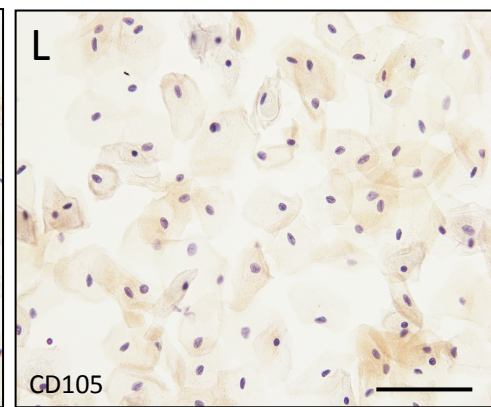

Supplement: Supplementary file 3 — Supplementary Material 3: Suppl. Figure 3 Shows expression of different biological markers in neonatal uterine blood. Immunocytochemical staining of different biological markers (A-H) and epithelial mesenchymal stem cells (eMSCs) (I-L) in prospectively collected neonatal uterine blood (NUB). Squamous cells in NUB showed strong positive cytoplasmic reaction to PGR (D), Gd-A (G), and SUSD2 (I) and weak or no staining for other markers (EpCAM, CD10, ER, PRL, IGFBP1, α-SMA) and/or other eMSCs markers (PDGFRβ, CD90, CD105). The respective abbreviation for each of these biological markers is mentioned in the text. [file 12958_2023_1099_MOESM3_ESM.pdf]
