# Supplementary material for: Is neonatal uterine bleeding responsible for early-onset endometriosis?
Source: Reprod Biol Endocrinol. 2023 Jun 19;21:56. doi: 10.1186/s12958-023-01099-1 (PMC10278367; doi:10.1186/s12958-023-01099-1)
Supplement: Supplementary file 4 — Supplementary Material 4: Suppl. Table 1. Shows immunohistochemical staining rates of different biological markers in neonatal endometria. [file 12958_2023_1099_MOESM4_ESM.docx]

Suppl. Table 1. Immunohistochemical staining rates of different biological markers in neonatal endometria collected from 15 autopsy cases

| gland cells stromal cells  n (%) n (%) |
| --- |
| **EpCAM**  14 (93.4)  **CD10** 14 (93.4)  **ER** 10 (66.7) 12 (80)  **PGR** 12 (80) 8 (53.3)  **Ki-67** 9 (60) 8 (53.3)  **CD31**  14 (93.4)  **PRL** 10 (66.7) 9 (60)  **IGFBP1** 5 (33.4) 0 (0)  **Glycodelin-A**  15 (100)  **α-SMA** 15 (100)  **CD68** 14 (93.4)  **CD45** 14 (93.4)  **CD56**  11 (73.3)  **SUSD2**  0 (0)  **PDGFRβ** 0 (0)  **CD90** 15 (100)  **CD105** 15 (100) |

EpCAM, epithelial cell adhesion molecule, marker of gland cells; CD10, marker of stromal cells; ER, estrogen receptor; PGR, progesterone receptor; Ki-67, marker of cell proliferation; CD31, marker of vascular cells; PRL, prolactin, decidual marker; IGFBP1, insulin growth factor binding protein 1, decidual marker; Glycodelin-A, predecidual marker; α-SMA, alpha smooth muscle actin, predecidual marker; CD68, marker of macrophages; CD45, marker of pan-leukocytes; CD56, marker of natural killer cells; SUSD2, Sushi domain containing 2, PDGFRβ, platelet-derived growth factor receptor-beta, CD90, CD105, all are different phenotypes of endometrial mesenchymal stem cells (eMSCs) marker.
